# Prevalence of the *Cladosporium cladosporioides* Species Complex in the Mycelia-Like Skin Crusts of Migratory Yellow-Throated Buntings (*Emberiza elegans*) in Korea

**DOI:** 10.1007/s11046-025-00935-9

**Published:** 2025-02-27

**Authors:** Seung-Kyung Lee, Se-Young Park, Hwa-Yeon Kang, Se-Jeong Han, Hyun-Young Nam, Chang-Yong Choi, Naomichi Yamamoto

**Affiliations:** 1 Department of Environmental Health Sciences, Graduate School of Public Health, Seoul National University, Seoul, Republic of Korea; 2 Institute of Health and Environment, Seoul National University, Seoul, Republic of Korea; 3 Department of Agriculture, Forestry and Bioresources, College of Agriculture and Life Sciences, Seoul National University, Seoul, Republic of Korea; 4 School of Biological Sciences, College of Natural Sciences, Seoul National University, Seoul, Republic of Korea

**Keywords:** Buntings, Fungal flora, Phaeohyphomycosis, Mycoses, Migratory birds, Wild passerines

## Abstract

Understanding the ecological characteristics and environmental factors of migratory songbirds is essential for their conservation as well as pathogen management that may cross ecological and political boundaries. In this study, we conducted a bird trapping and banding survey and report on fungal DNA detected from birds with putative fungal skin infections. We analyzed the mycobiome of mycelia-like skin crusts of the yellow-throated bunting (*Emberiza elegans*), a common migratory songbird with declining population in Korea, using DNA metabarcoding targeting the internal transcribed spacer 1 (ITS1) region, the actin (ACT) gene, and the translation elongation factor 1 − α (TEF) gene. Our analysis revealed that *Cladosporium* was the predominant genus (~ 60% sequence reads) in fungal mycelia-like tissues on the skins of yellow-throated buntings and detected a large number of DNA sequences similar to those of species belonging to the *Cladosporium cladosporioides* species complex. This is the first study to report possible infection in buntings by *Cladosporium*, including species known to infect humans and other animals. Further research on the causal relationship between birds and fungi is needed for pathogen management and conservation of Asian songbirds along the migration flyway.

**Supplementary Information:**

The online version contains supplementary material available at 10.1007/s11046-025-00935-9.

## Introduction

The decline in migratory bird populations is a major concern for both birds and ecosystems, particularly in the East Asian Flyway [1–3]. Wild bird populations are facing numerous threats, including habitat loss and changes [4–6], climate changes affecting migration and breeding timing [7, 8], illegal trapping and killing [9, 10], and infectious diseases [11]. Land birds, particularly songbirds including buntings (*Emberiza* spp.), are affected, experiencing a significant population decline [12]. For example, the rustic bunting (*Emberiza rustica*) population declined significantly, 32–91% decrease within 10 years, showing the trend of population collapse leading to a change in its International Union for Conservation of Nature (IUCN) Red List status from Least Concern to Vulnerable in a very short period [13, 14]. Notably, buntings are declining in population across various Asian regions [10, 12, 15].

The yellow-throated bunting (*Emberiza elegans*) is a migratory songbird regularly passing through the Korean Peninsula. Although the bunting exhibits a migratory behavior, the species is present throughout the year in the Korean Peninsula as a partial migrant [16]. The yellow-throated bunting is known to take East Asia as its flyways, spends winter in the Korean Peninsula, southern China, Taiwan, and Japan, and breeds in the Korean Peninsula, Far East Russia, and northern China [16–18]. The bunting is categorized into Least Concern by IUCN without clear evidence of a decline in population or the presence of substantial threats [19], but in South Korea, a population decrease has been evidenced by several studies [12, 20].

Understanding the microbiome on an animal’s skin can provide valuable information for the conservation of the animal species [21, 22]. Furthermore, it allows for the identification and tracing of the origins of diseases that may affect the health of humans and animals [21, 23]. Migratory birds are known as possible carriers and reservoirs for various infectious diseases caused by fungi, bacteria, protozoa, and so on, which can be transmitted to humans across the ecological or political barriers [24–26]. These diseases include the avian influenza virus, duck plague virus, *Salmonella* spp., *Escherichia coli*, *Mycobacterium avium*, as well as pathogenic fungi such as *Aspergillus* spp. and *Candida* spp. [26]. For example, aspergillosis, a mycosis caused by *Aspergillus* spp., is known to occur in several migratory bird species, such as black-headed gulls (*Larus ridibundus*) [27] and white storks (*Ciconia ciconia*) [28]. Therefore, understanding of pathogens in migratory birds is essential to safeguard animal health and public health and to improve our readiness for associated disease prevention and mitigation.

Among mycoses, phaeohyphomycosis is a rare fungal disease caused by dark-pigmented, dematiaceous fungi that can lead to infections to the central nervous system, skin, subcutis, and paranasal sinuses [29]. These mycoses exhibit various infection patterns, including systemic, cutaneous and corneal subcutaneous, and superficial infections [29, 30]. These fungal species are ubiquitous, being found in soil, plants, wood, and air. Occasionally, they exhibit rare opportunistic pathogenic behavior towards animals and humans [31, 32]. Moreover, these mycoses affect both domestic and wild animals, including amphibians, fish, reptiles, and birds [33, 34]. Furthermore, phaeohyphomycetes, or dematiaceous fungi, can cause infections in both immunocompromised and immunocompetent hosts [33, 35]. Several dematiaceous fungi, such as *Alternaria*, *Cladosporium*, *Exophiala,* and *Mycoleptodiscus*, are known to affect the health of both humans and animals [33, 34, 36–38].

Among them, *Cladosporium* is a cosmopolitan fungal genus with diverse ecological roles [39]. The species include saprobic environmental fungi, leaf endophytes, and pathogenic fungi to animals and plants that can be found in various environments, such as indoors, air, soil, and leaves [40–45]. Some species are pathogenic and can affect both humans and animals, causing health problems [45–47]. In particular, species within the *Cladosporium cladosporioides* species complex are known to cause infections in animals, such as skin infections in dogs [48], brain infections in cats [46], systemic infections in sheep [49], and skin infections in a giant panda [50].

**Fig. 1:**
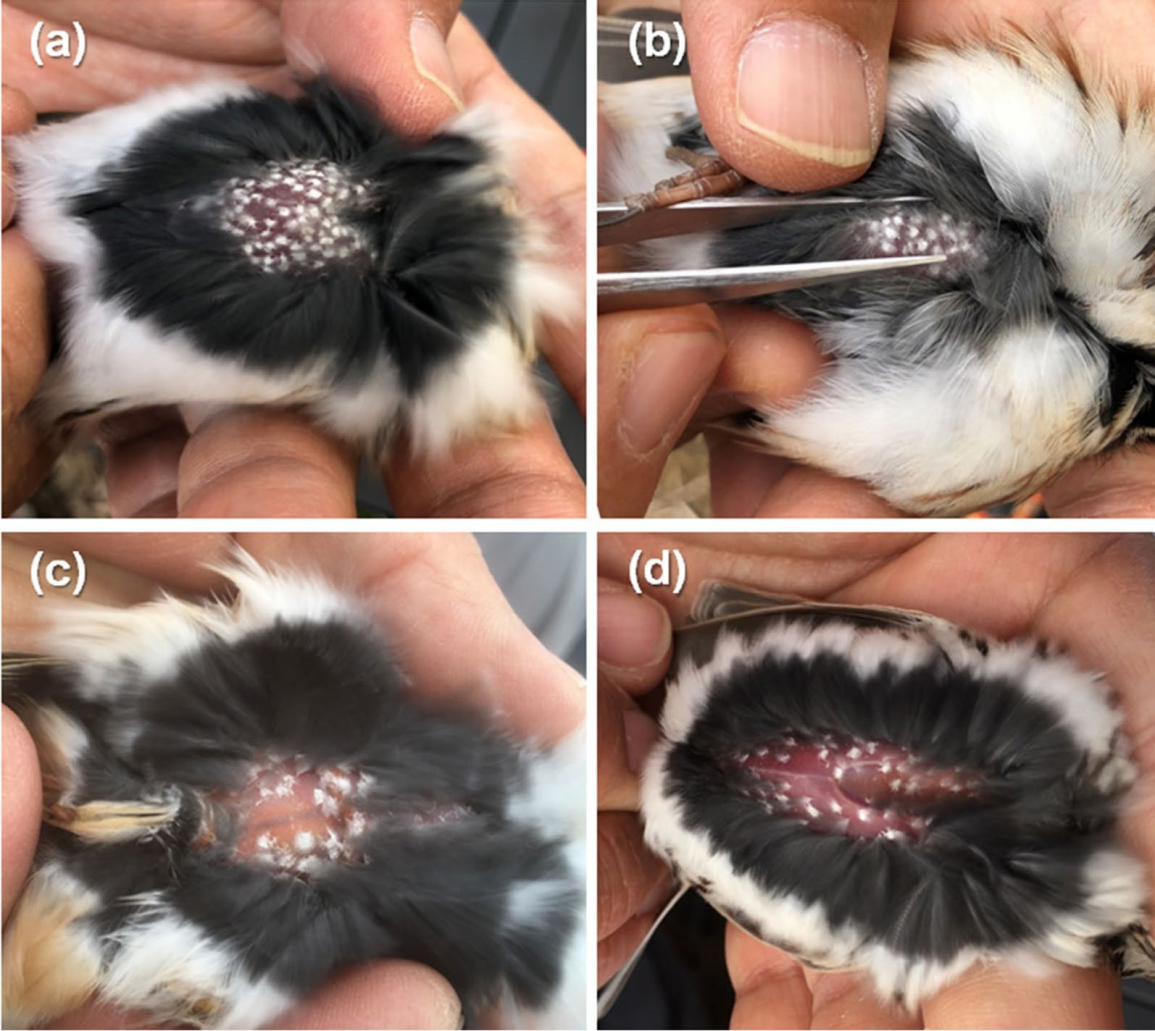
Putative skin fungal infections found on **a** a yellow-throated bunting (*Emberiza elegans*) at Haenam-gun (28 Dec 2021), **b** another yellow-throated bunting at Haenam-gun (28 Dec 2021), **c** a brambling (*Fringilla montifringilla*) on Daecheong Island (26 April 2021), and **d** a little bunting (*Emberiza pussila*) on Daecheong Island (2 May 2021), Korea. For a clear presentation of the skin fungal mycelia-like tissue, depicted as hyaline and circular spots, only abdomen skins are displayed here. The anterior parts (head) of all birds are positioned on the right side

During our recent bird trapping and banding survey conducted in Korea, we observed a significant number of wild migratory songbirds, including the yellow-throated bunting, exhibiting hyaline and circular fungal mycelia-like tissues on their skins (Fig. 1). Based on empirical evidence over the past two decades, it is estimated that these mycelia-like tissues found on the skin of migratory birds are increasing compared to the past. To our knowledge, there are no reports on heavy fungal mycelia-like tissues on the skins of migratory songbirds, and information about associated fungi as well as the trend of prevalence in this geographic region and flyway is lacking. Therefore, in this study, we aimed to report on the prevalence of wild bird species with heavy skin fungal mycelia-like tissues obtained by our survey in Korea, and characterize the mycobiome of those of yellow-throated buntings, and identify fungal species using high-throughput amplicon sequencing of three fungal DNA barcode markers: the internal transcribed spacer 1 (ITS1) region, the actin (ACT) gene, and the translational elongation factor 1-⍺ (TEF) gene.

## Materials and Methods

### Study Sites

A bird trapping and banding survey was conducted at multiple sites during the spring and fall migration seasons from 2021 to 2023 (Fig. S1). Daecheong Island (Ongjin-gun, Incheon; 37° 50′ N, 124° 42′ E) was the main site operated in April, May, October, and November in 2022 and 2023 (Permit No. 2022–32, 2023–40), while there were occasional investigations in different sites: Gwangju-si (Gyeonggi-do; 37° 18′ N, 127° 18′ E) in October 2021 (Permit No. 2021–1), and Jeombong mountain (Inje-gun, Gangwon-do; 38° 02′ N, 128° 28′ E) in October 2022 (Permit No. 433000085202200003). In addition, we also captured wintering birds at Haenam-gun (Jeollanam-do; 34° 37′ N, 126° 37′ E) in December 2021 (Permit No. 2021–7), Hwaseong-si (Gyeonggi-do; 37° 15′ N, 126° 48′ E) in December 2021, December 2022, and January and March 2023 (Permit No. 2021–3, 2021–12, 2022–8), Boryeong-si (Chungcheongnam-do; 36° 25′ N, 126° 31′ E) in January 2022 (Permit No. 2022–2), Jeju-si in Jeju island (33° 53′ N, 126° 67′ E) in January 2022 (Permit No. 2020–5), Seogwipo-si in Jeju island (33° 25′ N, 126° 31′ E) in February 2022 (Permit No. 2022–7), and Gwangju-si in December 2022 (Permit No. 2022–1). Lastly, we carried out the banding investigations during the summer at Hanam-si (Gyeonggi-do; 37° 29′ N, 127° 13′ E) in June 2022 (Permit No. 404000085202200001), Jeombong mountain in January 2022 (Permit No. 433000085202200003), and Gwangju-si in June 2023 (Permit No. 2023–1).

### Sampling Procedure

Yellow-throated buntings and other migratory birds were captured using mist nets during the daytime and were individually banded with aluminum rings. We identified the species, sex, and age based on their morphological features including plumages and feathers, skull ossification levels, and molting conditions, and then, we also measured wing length, tail length, body mass, and fat score [51]. In particular, each bird was inspected for the skin conditions during the visual assessment of body conditions and fat score [52, 53]. Each captured bird was placed on its side on the researcher’s palm and the bird’s neck was positioned between the second and third fingers of the hand to prevent escape. Then, both legs were gently pulled together while the ventral and side contour feathers were blown aside to expose their skins [52, 53]. For the yellow-throated bunting, when prominent hyaline, circular fungal mycelia-like tissues, possibly indicative of fungal infection was visually confirmed on its bare skin (Fig. 1), multiple (usually 5–20 based on availability) thin sheets of mycelia-like tissue were carefully removed by picking them up with ethanol-sterilized forceps and stored in a sterilized cryovial under − 20 °C (Table S1).

All survey processes followed a constant effort survey protocol of the Korean National Park Research Institute [51], and the required permissions for bird capturing, banding, and sample collection were obtained from the local governments (see “Study sites” for detailed permit information). All captured and examined birds were released in 30 min safely.

### DNA Extraction

Since the amount of each sample collected was less than 0.001 g, all samples were used for DNA extraction. An AccuPrep® Tissue and Blood Kit (Bioneer Corporation, Daejeon, South Korea) was used for DNA extraction, following the manufacturer’s instruction with a modification of adding 0.3 g of glass beads with a diameter of 0.1 mm and 0.1 g of glass beads with a diameter of 0.3 mm into a sterilized tube, which was homogenized using a beadbeater-24 (Biospec Products, Inc., Bartlesville, OK, USA) [54]. Finally, DNA was eluted into 50 µl of TE. The extracted DNA was stored at − 80 °C until further processing.

### DNA Sequencing

The extracted DNA was PCR amplified using fungal specific primers targeting each region (Table S2). ITS1F and ITS2 were used for amplifying the ITS1 region [55], ACT-512F and ACT-783R were used for amplifying ACT, and EF1-728F and EF1-986R were used for amplifying TEF [56]. Each primer was tagged with a MiSeq adapter (Illumina, Inc., San Diego, CA, USA). Each PCR mixture (25 µl) consisted of 12.5 µl of 2 × PCR Solution Premix Taq (Takara Bio Inc., Otsu, Shiga, Japan) and 1 µl of extracted DNA. The concentration of each primer was 0.3 µM. The PCR conditions used for each primer set were as follows: ITS1, initial denaturation at 94 °C for 5 min, 35 cycles of 94 °C for 30 s, 55 °C for 30 s, 72 °C for 60 s and final elongation at 72 °C for 10 min; ACT, initial denaturation at 94 °C for 5 min, 40 cycles of 94 °C for 45 s, 52 °C for 30 s, and 72 °C for 90 s, and final elongation at 72 °C for 5 min; TEF, initial denaturation at 94 °C for 5 min, 40 cycles of denaturation at 94 °C for 45 s, annealing at 60 °C for 30 s, and 72 °C for 90 s, and final elongation at 72 °C for 5 min. The amplification product was checked with 1% agarose gel (BIOFACT, Daejeon, South Korea) with gel electrophoresis. During the PCR processes, the TEF region for samples 1 and 9 failed to amplify in numerous attempts; therefore, they were excluded from further procedures (Table S1). After PCR clean-up processes, the DNA was indexed using a Nextera XT Index Kit v2 (Illumina). The thermal cycle for the index PCR was as follows: initial denaturation at 95 °C for 3 min, 8 cycles of 95 °C for 30 s, 55 °C for 30 s, and 72 °C for 30 s, and final elongation at 72 °C for 5 min. Finally, PCR products were purified using AMPure XP beads (Beckman Coulter), and quantification was performed using a Quant-iT PicoGreen dsDNA reagent kit (Life Technologies, Carlsbad, CA, USA). Then, the samples were normalized and pooled with internal control PhiX (30%) using 2 × 300 bp paired-end sequencing in v3 600 cycle kit reagent cartridge, sequenced by an Illumina MiSeq system. Raw sequences were submitted to Sequence Read Archive database of National Center for Biotechnology Information (NCBI) under the BioProject accession number PRJNA1056340.

### Bioinformatics

Using DADA2 [57], in R version 4.2.1 [58], primer sequences were removed from raw sequence reads, and the sequences were quality checked, trimmed, merged, and chimeric sequences were removed. Then, denoised and clustered into Amplicon Sequence Variants (ASVs). For quality control, the sequences that had maximum error rates greater than 1 nt and/or read lengths less than 200 nt for ITS1 and TEF, and 180 nt for ACT were removed. During the quality control processes, for the ACT region of samples 5 and 6, and the TEF region of sample 6, no sequences remained for further analysis. Consequently, they were excluded from the analysis (Table S1). For ITS1, generated ASVs were blasted against the Fungal Internal Transcribed Spacer RNA (ITS) RefSeq Targeted Loci Project database (http://www.ncbi.nlm.nih.gov/bioproject/PRJNA177353), downloaded from NCBI on March 6, 2023 using NCBI BLASTN 2.13.0 + [59]. For TEF and ACT, the sequences were blasted against the NCBI nucleotide collection (nt) database on April 5 and 10, 2023 respectively. The taxonomy of the sequences was determined by analyzing the blast results using FHiTHINGs v.1.5. [60], with a maximum error rate of 0.001.

### Data Analysis

As there was significant variance in the abundance of sequence reads across the samples (Table S1), without rarefying the samples, instead, we used relative read abundance for further analysis. Firstly, the relative abundance of genera in each sample was calculated and visualized using “ggplot2” [61] package in R version 4.2.1 [58]. The heatmap showing the relative abundance of ASVs identified as *Cladosporium* spp. in each marker gene was generated using “pheatmap” package [62]. For phylogenetic analysis, the sequences of ASVs that were identified as *Cladosporium* spp. and the type sequences of *Cladosporium* spp. were aligned using a multiple sequence alignment tool based on the MUSCLE algorithm [63] at the EMBL-EBI website. Then, using IQ-TREE version 2.3.4 [64], the phylogenetic trees were constructed by the Maximum Likelihood method with 1000 bootstraps [65] for each DNA marker (i.e., ITS1, ACT, and TEF). The best-fit substitution model was determined based on Bayesian Information Criterion [66]. As the outgroup of the phylogenetic trees, the *Cercospora beticola* sequences were used. For both ASVs and type sequences, only the sequences in the region between forward and reverse primers were extracted and used. A list of species included and their type sequence accession number information can be found in Table S3. Visualization of phylogenetic trees was conducted using Interactive Tree Of Life (iTOL) version 6.9 [67].

## Results

### Number of Birds Confirmed with Fungal Mycelia-Like Tissues on Skin

A total of 3,272 individuals representing 95 bird species were captured and examined during routine bird banding surveys. Distinctive fungal mycelia-like tissues on their skins were confirmed in 32 individuals, constituting 0.98% of the total. The fungal mycelia-like tissues on skins were detected in 11 songbird species. Among these, yellow-throated buntings showed a significant positivity rate of fungal mycelia-like tissues (17 out of 133 birds, 12.8%), while two other migratory buntings exhibited low positivity rates: little (*Emberiza pusilla*; 1 out of 656 examined, 0.2%) and rustic buntings (*E. rustica*; 1 out of 157, 0.6%). Other species with varying positivity rates included Oriental reed warblers (*Acrocephalus orientalis*; 1 out of 20, 5.0%), red-flanked bluetails (*Tarsiger cyanurus*; 1 out of 21, 4.8%), warbling white-eyes (*Zosterops japonicus*; 1 out of 30, 3.3%), bramblings (*Fringilla montifringilla*; 3 out of 56, 5.4%), blue-and-white flycatchers (*Cyanoptila cyanomelana*; 2 out of 6, 33.3%), Asian brown flycatchers (*Muscicapa dauurica*; 1 out of 32, 3.1%), Stejneger’s stonechats (*Saxicola stejnegeri*; 3 out of 28, 10.7%), and bull-headed shrikes (*Lanius bucephalus*; 1 out of 13, 7.7%) (Table 1). After collecting the thin films of fungal mycelia-like tissue, no signs of lesions or other indications of cutaneous/subcutaneous infection remained in the collected skin areas.

**Table 1:** Proportion of birds in Korea with fungal mycelia-like tissues on their skins

Scientific name (common name)	Type	No. of birds examined (A)	No. of birds with mycelia-like crusts (B)	% Proportion (B/A)
*Acrocephalus orientalis* (Oriental reed warbler)	Migrant	20	1	5.0
*Cyanoptila cyanomelana* (blue-and-white flycatcher)	Migrant	6	2	33.3
*Emberiza elegans* (yellow-throated bunting)	Partial migrant	133	17	12.8
*Emberiza pusilla* (little bunting)	Migrant	656	1	0.2
*Emberiza rustica* (rustic bunting)	Migrant	157	1	0.6
*Fringilla montifringilla* (brambling)	Migrant	56	3	5.4
*Lanius bucephalus* (bull-headed shrike**)**	Resident	13	1	7.7
*Muscicapa dauurica* (Asian brown flycatcher)	Migrant	32	1	3.1
*Saxicola stejnegeri* (Stejneger’s stonechat)	Migrant	28	3	10.7
*Tarsiger cyanurus* (red-flanked bluetail)	Migrant	21	1	4.8
*Zosterops japonicus* (warbling white-eye)	Partial migrant	30	1	3.3

### Skin Crust Mycobiome

**Fig. 2:**
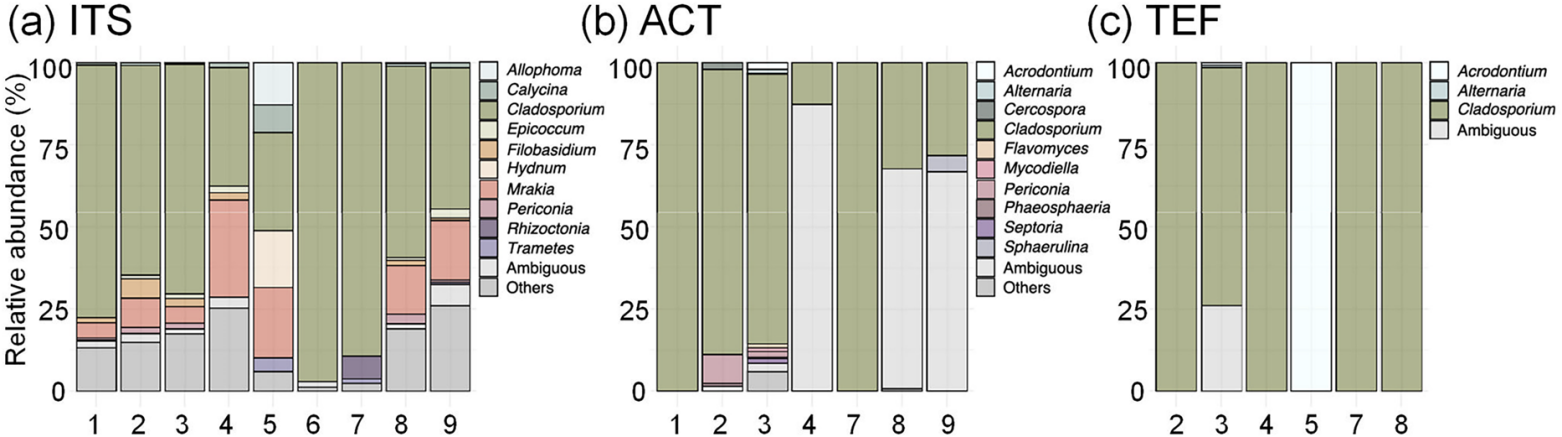
Relative abundance of the top 10 fungal genera identified by **a** ITS1, **b** ACT, and **c** TEF sequences

From the sequencing results of the ITS1 region, it was observed that the genus *Cladosporium* was most dominant across all samples (average 62.7% of relative read abundance for the genus in all samples; Fig. 2a). Additionally, the genus *Mrakia* (11.4% of relative abundance) was also identified with high relative read abundance (Fig. 2a). ACT gene sequencing results also revealed that *Cladosporium* was the predominant fungus in most samples (Fig. 2b). Similarly, TEF gene sequencing results revealed that *Cladosporium* was dominant in most samples, except for the sample 5 (Fig. 2c).

### Phylogenetic Analysis of Cladosporium Sequences

**Fig. 3:**
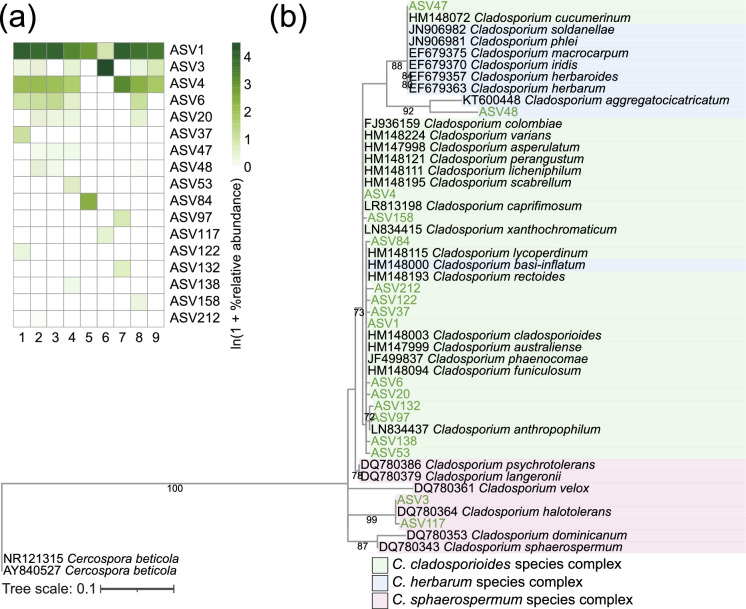
**a** Relative abundance of ASVs identified as *Cladosporium* spp. based on the sequences of the ITS1 region. **b** Phylogenetic tree containing type strains (in black text) and ASVs identified as *Cladosporium* spp. in this study (in green text) based on the sequences of the ITS1 region. *Cercospora beticola* was used as the outgroup of the tree. The best-fit model identified using the Bayesian Information Criterion was K2P + R2. Only bootstrap values above 70% are shown in the nodes

**Fig. 4:**
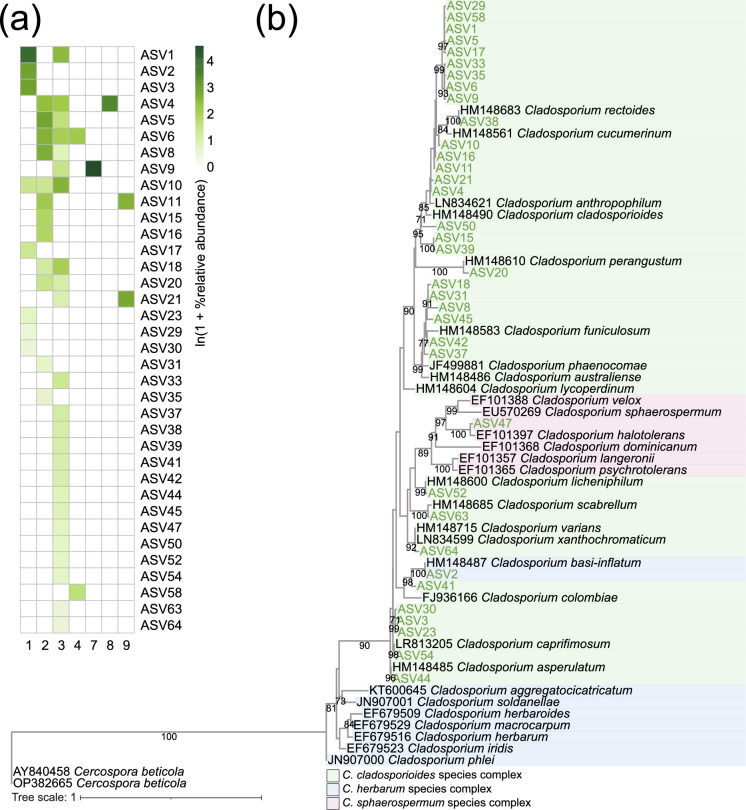
**a** Relative abundance of ASVs identified as *Cladosporium* spp. based on the sequences of the ACT gene. **b** Phylogenetic tree containing type strains (in black text) and ASVs identified as *Cladosporium* spp. in this study (in green text) based on the sequences of the ACT gene. *Cercospora beticola* was used as the outgroup of the tree. The best-fit model identified using the Bayesian Information Criterion was HKY + F + I + G4. Only bootstrap values above 70% are shown in the nodes

**Fig. 5:**
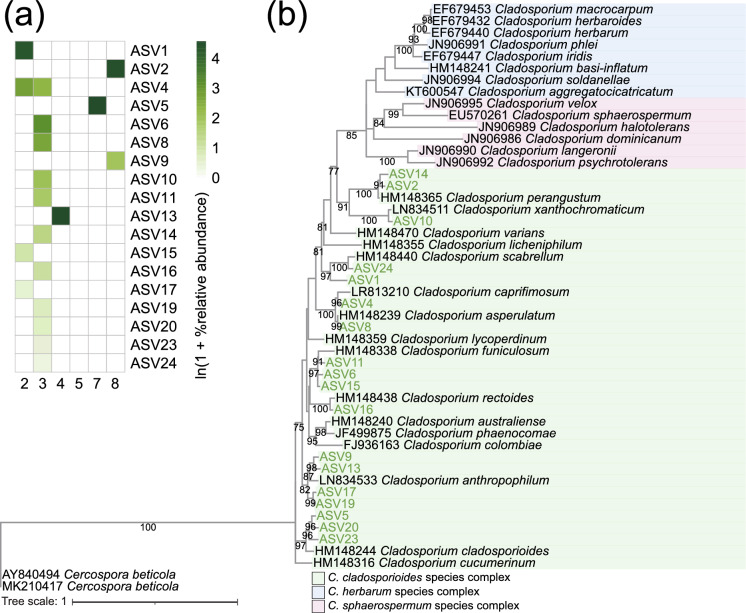
**a** Relative abundance of ASVs identified as *Cladosporium* spp. based on the sequences of the TEF gene. **b** Phylogenetic tree containing type strains (in black text) and ASVs identified as *Cladosporium* spp. in this study (in green text) based on the sequences of the TEF gene. *Cercospora beticola* was used as the outgroup of the tree. The best-fit model identified using the Bayesian Information Criterion was TIM2e + I + G4. Only bootstrap values above 70% are shown in the nodes

The phylogenetic trees were constructed with the sequences from the type specimen of *Cladosporium* species. According to the phylogenetic analysis based on the ITS region, the ASVs identified as *Cladosporium* spp. were not fully resolved taxonomically at the species level, and furthermore, the branching pattern of the phylogenetic tree did not reflect the phylogenetic pattern of the three species complexes of *Cladosporium* (Fig. 3). In contrast, phylogenetic analysis based on sequences of the ACT and TEF genes shows clear genetic differentiation among *Cladosporium* species, and the branching patterns of the phylogenetic trees also clearly indicated the phylogenetic patterns of the three species complexes of *Cladosporium* (Figs. 4 and 5). For ACT, the majority of ASVs identified as *Cladosporium* spp. were clustered into the *C. cladosporioides* species complex (Fig. 4). Similarly, for TEF, all ASVs identified as *Cladosporium* spp. were clustered into the *C. cladosporioides* complex (Fig. 5). These results indicate that most of the fungi detected from birds with fungal mycelia-like tissue formed are *Cladosporium* species belonging to the *C. cladosporioides* species complex. Raw sequence data of ASVs assigned as *Cladosporium* species are shown in Data S1–S3.

## Discussion

The skins of 11 migratory songbird species, including yellow-throated buntings, exhibited multiple prominent hyaline and circular fungal mycelia-like tissue. The extent of the tissue varied among individuals, but, in general, the fungal mycelia-like tissues were distributed throughout the skins of the entire main bodies. The absence of signs of lesions or cutaneous/subcutaneous fungal infection on skins suggests that the possible fungal infection is primarily confined to the superficial layers. Investigation of the mycobiome from the yellow-throated buntings by DNA metabarcoding of multiple loci (ITS1, ACT, and TEF) revealed that the genus *Cladosporium* was dominant in all samples. We also showed that the use of multiple loci is essential for species-level discrimination of *Cladosporium* in the mycobiome of the birds. Furthermore, most of the detected *Cladosporium* species were found to belong to the *C. cladosporioides* species complex, which may cause phaeohyphomycosis in the buntings.

### Skin Crust Mycobiome of Yellow-Throated Buntings with Fungal Mycelia-Like Tissues

Sequencing of the ITS1 region revealed that *Cladosporium* was the most abundant genus in the fungal mycelia-like tissues found on the skin crusts of yellow-throated buntings (Fig. 2a). *Cladosporium* is ubiquitous in various environments such as air, soil, and indoors [40, 41], and *C. cladosporioides* has been reported to be present in the feathers of migratory birds and related environments [68, 69]. Moreover, *C. cladosporioides* has been reported to be found in nests of the blue tit (*Cyanistes caeruleus*) and great tit (*Parus major*) [70, 71]. Furthermore, in addition to *Cladosporium*, the genus *Mrakia* was also frequently detected in the skin crust mycobiome. *Mrakia* has been reported to be detected in the feathers of the barnacle goose (*Branta leucopsis*) [72].

### Resolution of Cladosporium Species Identification by DNA Sequencing

The ITS region is commonly used as a primary DNA marker to identify *Cladosporium* [42, 73, 74], but our results suggest that sequencing the ACT and TEF genes is essential for identifying *Cladosporium* at the lower taxonomic levels. Our result of the phylogenetic tree of *Cladosporium* based on the sequences of the ITS region indicates that it is nearly impossible to identify *Cladosporium* at the species level (Fig. 3). In fact, several studies have reported that the ITS region has limited resolution for identifying *Cladosporium* at the species level [73, 74]. Analysis of the *C. herbarum* species complex also reveals that sequence differences in the ITS region between species are so small that species-level identification is nearly impossible [74]. On the other hand, our study confirmed that *Cladosporium* can be identified in more detail at the lower taxonomic levels by sequencing the ACT or TEF genes (Figs. 4 and 5). This is consistent with previous literature reporting that inclusion of additional genes such as ACT and TEF is essential for accurately identifying *Cladosporium* at the species level [74, 75].

### Cladosporium cladosporioides Species Complex as the Major Fungal Group in the Skin Crust Mycobiome of Birds with Fungal Mycelia-Like Tissues

According to our phylogenetic tree analysis of ACT and TEF genes, most of the ASVs identified as *Cladosporium* were found to belong to the *C. cladosporioides* species complex (Figs. 4 and 5). It is well known that *Cladosporium* species such as *C. cladosporioides*, *Cladosporium anthropophilum,* and *Cladosporium oxysporum* belonging to this species complex cause/are associated with infections in humans and animals [46, 47, 75–77]. Furthermore, *C. cladosporioides* species complex is also known to cause phaeohyphomycosis in animals [46, 50, 75]. Symptoms of *C. cladosporioides* species complex infection are diverse and can affect different parts of animals’ body [46]. For instance, in giant panda, it is known to cause cutaneous infection on the nose [50], while in merino sheep, it is known to cause systemic infections affecting the lung, abomasum, liver, kidneys, and heart [49]. In dogs, it is known to result in respiratory infections in the mediastinal lymph nodes [48]. In our study, a large number of ASVs associated with the *C. cladosporioides* species complex were detected from the skin crusts of buntings with fungal mycelia-like tissues. Similar to the previous studies, the *Cladosporium* spp. detected in this study are thought to be infecting the buntings and having some kind of health impacts on them.

### Migratory Birds and Their Interactions with Fungal Diseases

Understanding the microbiomes and mycopathological relationships on wildlife is informative and essential for the health and conservation of the species as well as the management of potential zoonotic diseases [22]. Migratory birds are recognized as potential carriers and reservoirs of pathogenic fungi that can infect humans, domestic animals, and themselves [24, 25, 68]. Our findings highlight the dominance of the *C. cladosporioides* species complex in mycelia-like skin crusts of yellow-throated buntings and possibly other migratory songbirds in Korea. However, the causal relationship and the impact of fungal infections with the overspread mycelia-like tissues found on the bird skins remains unknown. Nevertheless, it is known that these infectious and opportunistic pathogenic fungi can affect both immunocompromised and immunocompetent hosts [78]. While a study has indicated that migratory birds play a limited role in transmitting pathogens and diseases to humans, the mechanisms, directions of transmission, and causal relationships between wild birds and humans remain uncertain [26]. Therefore, further analysis of these etiological relationships is necessary. Furthermore, during our banding survey, we identified mycelia-like skin crusts in 11 bird species, including nine obligated migratory species, which are also important components of the East Asian Flyways, and species facing a conservation crisis (e.g., rustic bunting and little bunting) [1, 13] even though they exhibited the relatively low prevalence. This emphasizes the need for a more comprehensive understanding of potential phaeohyphomycosis across various migratory bird species.

## Conclusions

The dominance of the *C. cladosporioides* species complex in skin crusts of yellow-throated buntings raises concerns about its potential role in causing phaeohyphomycosis in the songbirds across their migratory flyway. The causal relationship between the *C. cladosporioides* species complex and wild migratory birds, including yellow-throated buntings, needs further research. For instance, the investigation of mycobiome on healthy skin, and consideration of birds’ health status would provide valuable insights into potential mycoses affecting bird species [79, 80]. An understanding of the mycobiome in migratory birds would provide insights into their fundamental ecological characteristics and, at the same time, help manage potential infectious diseases [21]. Therefore, we propose to conduct further research on mycoses and causative pathogens in wild migratory songbirds. This information is essential not only for the conservation of Asian songbirds, which are experiencing population declines, but also for gaining fundamental knowledge of fungi-bird relationships, particularly in mycopathological contexts.

## Supplementary Information

Below is the link to the electronic supplementary material.

Supplementary file1 (PDF 778 KB)
