# Supplementary material for: Prevalence of the Cladosporium cladosporioides Species Complex in the Mycelia-Like Skin Crusts of Migratory Yellow-Throated Buntings (Emberiza elegans) in Korea
Source: Mycopathologia. 2025 Feb 27;190(2):28. doi: 10.1007/s11046-025-00935-9 (PMC11868249; doi:10.1007/s11046-025-00935-9)
Supplement: Supplementary file 1 — Supplementary file1 (PDF 778 KB) [file 11046_2025_935_MOESM1_ESM.pdf]

Supplementary Information of:

Prevalence of the *Cladosporium cladosporioides* species complex in the mycelia-like skin crusts of migratory yellow-throated buntings (*Emberiza elegans*) in Korea

Seung-Kyung Lee[1,2], Se-Young Park[3], Hwa-Yeon Kang[3], Se-Jeong Han[3], Hyun-Young Nam[4], Chang-Yong Choi[3,*], Naomichi Yamamoto[1,2,*]

[1]Department of Environmental Health Sciences, Graduate School of Public Health, Seoul National University, Seoul, Republic of Korea

[2]Institute of Health and Environment, Seoul National University, Seoul, Republic of Korea

[3]Department of Agriculture, Forestry and Bioresources, College of Agriculture and Life Sciences, Seoul National University, Seoul, Republic of Korea

[4]School of Biological Sciences, College of Natural Sciences, Seoul National University, Seoul, Republic of Korea

*Co-corresponding authors

Chang-Yong Choi, E-mail: sub95@snu.ac.kr

Naomichi Yamamoto, E-mail: nyamamoto@snu.ac.kr

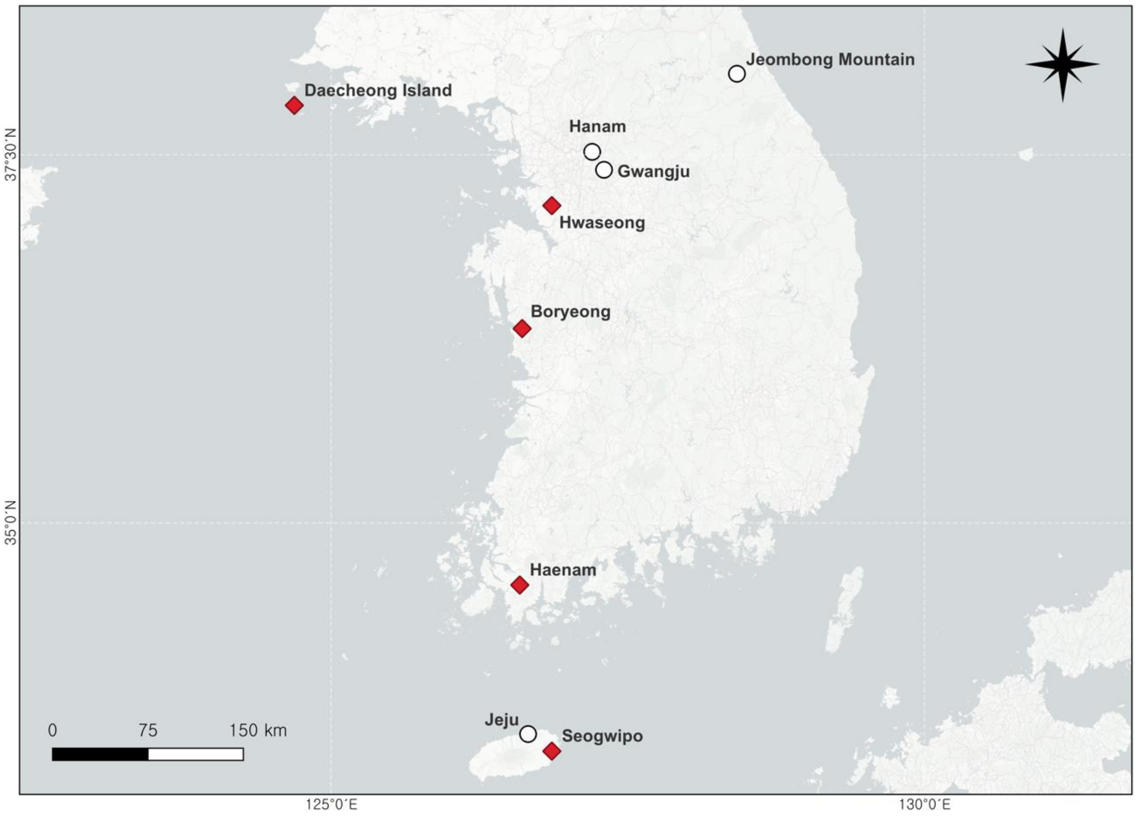

Fig. S1 Banding survey locations. White circles indicate the banding survey points, and the red diamonds locate sites where fungal mycelia-like structure were confirmed on bird skin

Table S1 Sample metadata and BioSample accession number. All sample data are deposited under the BioProject accession number PRJNA1056340 of NCBI

Sample number	ID	Host species	Sex	Age	Sampling date (YYYY/MM/DD)	Sampling location	ITS	ACT	TEF	Raw sequence reads			Sequence reads after quality control processes		
										ITS	ACT	TEF	ITS	ACT	TEF
**1**	021-09826	*Emberiza elegans*	M	A	2021/12/28	Haenam	SAMN39098277	SAMN39098286	NA [a]	58,147	161,531	NA [a]	31,089	19,313	NA [a]
**2**	021-09830	*Emberiza elegans*	F	1W	2021/12/28	Haenam	SAMN39098278	SAMN39098287	SAMN39098295	110,367	145,542	57,868	58,180	10,933	2,922
**3**	021-09843	*Emberiza elegans*	M	1W	2021/12/29	Haenam	SAMN39098279	SAMN39098288	SAMN39098296	65,282	90,827	62,564	34,182	3,250	1,718
**4**	021-09868	*Emberiza elegans*	M	1W	2022/01/19	Boryeong	SAMN39098280	SAMN39098289	SAMN39098297	77,666	154,343	51,198	43,487	582	73
**5**	021-09785	*Emberiza elegans*	M	A	2021/12/28	Haenam	SAMN39098281	SAMN39098290 [b]	SAMN39098298	52,843	163,721	72,071	2,319	0	811
**6**	021-09786	*Emberiza elegans*	M	1W	2021/12/28	Haenam	SAMN39098282	SAMN39098291 [b]	SAMN39098299 [b]	66,176	70,192	49,764	22,906	0	0
**7**	021-09794	*Emberiza elegans*	M	A	2021/12/28	Haenam	SAMN39098283	SAMN39098292	SAMN39098300	53,618	51,483	103,379	14,302	1,396	506
**8**	021-09796	*Emberiza elegans*	F	A	2021/12/28	Haenam	SAMN39098284	SAMN39098293	SAMN39098301	52,416	62,968	123,802	27,687	2,802	2,133
**9**	021-09863	*Emberiza elegans*	M	1W	2022/01/18	Boryeong	SAMN39098285	SAMN39098294	NA [a]	46,084	112,415	NA [a]	22,791	2,020	NA [a]

Abbreviation: M, male; F, female; A, adult; W, week; NA, not available

[a] Samples for which PCR amplification has been attempted multiple times but failed

[b] Samples for which no sequences remained for further data analysis after the quality control process

Table S2 Primer sets used in this study

Target region	Primer	Sequences (5'→3')	Reference
**ITS**	ITS1F	CTTGGTCATTTAGAGGAAGTAA	White et al. [1]
	ITS2	GCTGCGTTCTTCATCGATGC	
**ACT**	ACT-512F	ATGTGCAAGGCCGGTTTCGC	Carbone and Kohn [2]
	ACT-783R	TACGAGTCCTTCTGGCCCAT	
**TEF**	EF1-728F	CATCGAGAAGTTCGAGAAGG	
	EF1-986R	TACTTGAAGGAACCCTTACC	

Table S3 GenBank accession number of sequences used for phylogenetic tree analysis

Species		Strain	GenBank accession number		
			ITS	ACT	TEF
***Cladosporium cladosporioides* species complex**	*Cladosporium anthropophilum*	UTHSC DI-13-269	LN834437	LN834621	LN834533
	Cladosporium asperulatum	CBS 126340	HM147998	HM148485	HM148239
	Cladosporium australiense	CBS 125984	HM147999	HM148486	HM148240
	Cladosporium caprifimosum	FMR:16532	LR813198	LR813205	LR813210
	Cladosporium cladosporioides	CBS 112388	HM148003	HM148490	HM148244
	Cladosporium colombiae	CBS 274.80B	FJ936159	FJ936166	FJ936163
	Cladosporium cucumerinum	CBS 171.52	HM148072	HM148561	HM148316
	Cladosporium funiculosum	CBS 122129	HM148094	HM148583	HM148338
	Cladosporium licheniphilum	CBS 125990	HM148111	HM148600	HM148355
	Cladosporium lycoperdinum	CBS 574.78C	HM148115	HM148604	HM148359
	Cladosporium perangustum	CBS 12599	HM148121	HM148610	HM148365
	Cladosporium phaenocomae	CBS 128769	JF499837	JF499881	JF499875
	Cladosporium rectoides	CBS 125994	HM148193	HM148683	HM148438
	Cladosporium scabrellum	CBS 126358	HM148195	HM148685	HM148440
	Cladosporium varians	CBS 126362	HM148224	HM148715	HM148470
	Cladosporium xanthochromaticum	UTHSC DI-13-211	LN834415	LN834599	LN834511
***Cladosporium herbarum* species complex**	*Cladosporium aggregatocicatricatum*	CBS:140493	KT600448	KT600645	KT600547
	Cladosporium basi-inflatum	CBS 822.84	HM148000	HM148487	HM148241
	Cladosporium herbaroides	CBS:121626	EF679357	EF679509	EF679432
	Cladosporium herbarum	CBS:121621	EF679363	EF679516	EF679440
	Cladosporium iridis	CBS:138.40	EF679370	EF679523	EF679447
	Cladosporium macrocarpum	CBS:121623	EF679375	EF679529	EF679453
	Cladosporium phlei	CBS:358.69	JN906981	JN907000	JN906991
	Cladosporium soldanellae	CPC 13153	JN906982	JN907001	JN906994
***Cladosporium sphaerospermum* species complex**	*Cladosporium dominicanum*	CBS:119415	DQ780353	EF101368	JN906986
	Cladosporium halotolerans	CBS:119416	DQ780364	EF101397	JN906989
	Cladosporium langeronii	CBS:189.54	DQ780379	EF101357	JN906990
	Cladosporium psychrotolerans	CBS:119412	DQ780386	EF101365	JN906992
	Cladosporium sphaerospermum	CBS:193.54	DQ780343	EU570269	EU570261
	Cladosporium velox	CBS:119417	DQ780361	EF101388	JN906995
Cercospora	*Cercospora beticola*	CBS 116456	NR121315	AY840458	AY840494
		CPC 11557	AY840527	OP382665	MK210417

Data S1 DNA sequence reads of the ITS region assigned to *Cladosporium*

>ASV1
AAGTCGTAACAAGGTCTCCGTAGGTGAACCTGCGGAGGGATCATTACAAGTGAC
CCCGGTCTAACCACCGGGATGTTCATAACCCTTTGTTGTCCGACTCTGTTGCCTCC
GGGGCGACCCTGCCTTCGGGCGGGGGCTCCGGGTGGACACTTCAAACTCTTGCGT
AACTTTGCAGTCTGAGTAAACTTAATTAATAAATTAAAACTTTTAACAACGGATC
TCTTGGTTCTG
>ASV3
AAGTCGTAACAAGGTCTCCGTAGGTGAACCTGCGGAGGGATCATTACAAGTTGA
CCCCGGCCCTCGGGCCGGGATGTTCACAACCCTTTGTTGTCCGACTCTGTTGCCTC
CGGGGCGACCCTGCCTCCGGGCGGGGGCCCCGGGTGGACATTTCAAACTCTTGC
GTAACTTTGCAGTCTGAGTAAATTTAATTAATAAATTAAAACTTTCAACAACGGA
TCTCTTGGTTCTG
>ASV4
AAGTCGTAACAAGGTCTCCGTAGGTGAACCTGCGGAGGGATCATTACAAGTGAC
CCCGGTCTTACCACCGGGATGTTCATAACCCTTTGTTGTCCGACTCTGTTGCCTCC
GGGGCGACCCTGCCTTCGGGCGGGGGCTCCGGGTGGACACTTCAAACTCTTGCGT
AACTTTGCAGTCTGAGTAAACTTAATTAATAAATTAAAACTTTTAACAACGGATC
TCTTGGTTCTG
>ASV6
AAGTCGTAACAAGGTCTCCGTAGGTGAACCTGCGGAGGGATCATTACAAGTGAC
CCCGGTCTAACCACCGGGATGTTCATAACCCTTTGTTGTCCGACTCTGTTGCCTCC
GGGGCGACCCTGCCTTCGGGCGGGGGCTCCGGGTGGACACTTCAAACTCTTGCGT
AACTTTGCAGTCTGAGTAAACTTAATCAATAAATTAAAACTTTTAACAACGGATC
TCTTGGTTCTG
>ASV20
AAGTCGTAACAAGGTCTCCGTAGGTGAACCTGCGGAGGGATCATTACAAGTGAC
CCCGGTCTAACCACTGGGATGTTCATAACCCTTTGTTGTCCGACTCTGTTGCCTCC
GGGGCGACCCTGCCTTCGGGCGGGGGCTCCGGGTGGACACTTCAAACTCTTGCGT
AACTTTGCAGTCTGAGTAAACTTAATTAATAAATTAAAACTTTTAACAACGGATC
TCTTGGTTCTG
>ASV37
AAGTCGTAACAAGGTCTCCGTAGGTGAACCTGCGGAGGGATCATTACAAGTGAC
CCCGGTCTAACCACCGGGATGTTCATAACCCTTTGTTGTCCGACTCTGTTGCCTCC
GGGGCGACCCTGCCTTCGGGCGGGGGCTCCGGGTGGACACTTCAAACTCTTGCGT
AACTATGCAGTCTGAGTAAACTTAATTAATAAATTAAAACTTTTAACAACGGATC
TCTTGGTTCTG
>ASV47
AAGTCGTAACAAGGTCTCCGTAGGTGAACCTGCGGAGGGATCATTACAAGAACG
CCCGGGCTTCGGCCTGGTTATTCATAACCCTTTGTTGTCCGACTCTGTTGCCTCCG
GGGCGACCCTGCCTTCGGGCGGGGGCTCCGGGTGGACACTTCAAACTCTTGCGTA
ACTTTGCAGTCTGAGTAAACTTAATTAATAAATTAAAACTTTTAACAACGGATCT
CTTGGTTCTG
>ASV48
AAGTCGTAACAAGGTCTCCGTAGGTGAACCTGCGGAGGGATCATTATATGACGC
CCAGGCTTGTATAGCTGGGGACTGCTTAACCCTTTGTTTTCCGACTCTGTTGCCTC
CGGGGCGACCCTGCCTTCGGGCGGGGGCTCCGGGTGGACACTTCAAACTCTTGC
GTAACTTTGCAGTCTGAGTAAACTTAATTAATAAATTAAAACTTTTAACAACGGA
TCTCTTGGTTCTG

>ASV53
AAGTCGTAACAAGGTCTCCGTAGGTGAACCTGCGGAGGGATCATTACAAGTGAC
CCCGGTCTAACCACCGGGATGTTCATAACCCTTTGTCGTCCGACTCTGTTGCCTCC
GGGGCGACCCTGCCTTCGGGCGGGGGCTCCGGGTGGACACTTCAAACTCTTGCGT
AACTTTGCAGTCTGAGTAAACTTAATTAATAAATTAAAACTTTTAACAACGGATC
TCTTGGTTCTG
>ASV84
AAGTCTTAACAAGGTCTCCGTAGGTGAACCTGCGGAGGGATCATTACAAGTGAC
CCCGGTCTAACCACCGGGATGTTCATAACCCTTTGTTGTCCGACTCTGTTGCCTCC
GGGGCGACCCTGCCTTCGGGCGGGGGCTCCGGGTGGACACTTCAAACTCTTGCGT
AACTTTGCAGTCTGAGTAAACTTAATTAATAAATTAAAACTTTTAACAACGGATC
TCTTGGTTCTG
>ASV97
AAGTCGTAACAAGGTCTCCGTAGGTGAGCCTGCGGAGGGATCATTACAAGTGAC
CCCGGTCTAACCACCGGGATGTTCATAACCCTTTGTTGTCCGACTCTGTTGCCTCC
GGGGCGACCCTGCCTTCGGGCGGGGGCTCCGGGTGGACACTTCAAACTCTTGCGT
AACTTTGCAGTCTGAGTAAACTTAATTAATAAATTAAAACTTTTAACAACGGATC
TCTTGGTTCTG
>ASV117
AAGTCGTAACAAGGTCTCCGTAGGTGAACCTGCGGAGGGATCATTACAAGTTGA
CCCCGGCCCTCGGGCCGGGATGTTCACAACCCTTTGTTGTCCGACTCTGTTGCCTC
CGGGGCGACCCTGCCTCCGGGCGGGGGCCCCGGGTGGACATTTCAAACTCTTGC
GTAACTTTACAGTCTGAGTAAATTTAATTAATAAATTAAAACTTTCAACAACGGA
TCTCTTGGTTCTG
>ASV122
AAGTCGTAACAAGGTCTCCGTAGGTGAACCTGCGGAGGGATCATTACAAGTGAC
CCCGGTCTAACCACCAGGATGTTCATAACCCTTTGTTGTCCGACTCTGTTGCCTCC
GGGGCGACCCTGCCTTCGGGCGGGGGCTCCGGGTGGACACTTCAAACTCTTGCGT
AACTTTGCAGTCTGAGTAAACTTAATTAATAAATTAAAACTTTTAACAACGGATC
TCTTGGTTCTG
>ASV132
AAGTCGTAACAAGGTCTCCGTAGGTGAGCCTGCGGAGGGATCATTACAAGTGAC
CCCGGTCTAACCACCGGGATGTTCATAACCCTTTGTTGTCCGACTCTGTTGCCTCC
GGGGCGACCCTGCCTTCGGGCGGGGGCTCCGGGTGGACACTTCGAACTCTTGCGT
AACTTTGCAGTCTGAGTAAACTTAATTAATAAATTAAAACTTTTAACAACGGATC
TCTTGGTTCTG
>ASV138
AAGTCGTAACAAGGTCTCCGTAGGTGAACCTGCGAAGGGATCATTACAAGTGAC
CCCGGTCTAACCACCGGGATGTTCATAACCCTTTGTTGTCCGACTCTGTTGCCTCC
GGGGCGACCCTGCCTTCGGGCGGGGGCTCCGGGTGGACACTTCAAACTCTTGCGT
AACTTTGCAGTCTGAGTAAACTTAATTAATAAATTAAAACTTTTAACAACGGATC
TCTTGGTTCTG
>ASV158
AAGTCGTAATAAGGTCTCCGTAGGTGAACCTGCGGAGGGATCATTACAAGTGAC
CCCGGTCTTACCACCGGGATGTTCATAACCCTTTGTTGTCCGACTCTGTTGCCTCC
GGGGCGACCCTGCCTTCGGGCGGGGGCTCCGGGTGGACACTTCAAACTCTTGCGT
AACTTTGCAGTCTGAGTAAACTTAATTAATAAATTAAAACTTTTAACAACGGATC
TCTTGGTTCTG
>ASV212

AAGTCGTAACAAGGTCTCCGTAGGTGAACCCGCGGAGGGATCATTACAAGTGAC
CCCGGTCTAACCACCGGGATGTTCATAACCCTTTGTTGACCGACTCTGTTGCCTCC
GGGGCGACCCTGCCTTCGGGCGGGGGCTCCGGGTGGACACTTCAAACTCTTGCGT
AACTTTGCAGTCTGAGTAAACTTAATTAATAAATTAAAACTTTTAACAACGGATC
TCTTGGTTCTG

Data S2 DNA sequence reads of the ACT gene assigned to *Cladosporium*

>ASV1
CGGTGACGATGCGCCCAGAGCCGTTTTCCGTAAGTCCAAAGACACCTGTTTCGCC
CATCTCGCAATCCCAAGCTGACACTCTTCCTAGCTTCCATTGTCGGCAGACCCCG
TCACCATGGGTATGCATTCTCTCCGCGAGCCTCCCCATCGCGCACAGCCAGTTCT
AACCCCTCCGCAGTATCATGATCGGT
>ASV2
CGGTGACGATGCGCCCAGAGCCGTTTTCCGTAAGTCTGAAGACACCTGTTTCGCC
CATGCCACATTTCCGAGCTGACACCCATCATAGCTTCCATTGTCGGCAGACCCCG
TCACCATGGGTATGCACTCCCCCTCTCCCGCGTCTCTTCCCGCGCGCACTCAATAT
CTAACTCCGGCGCAGTATCATGATCGGT
>ASV3
CGGTGACGATGCGCCCAGAGCCGTTTTCCGTAAGTCTGAAGACACCTGTTTCGCC
CGTGCCAGAATTCCGAGCTGACACCATCCTAGCTTCCATTGTCGGCAGACCCCGT
CACCATGGGTATGCATCCTCCCCACCTCGCCCGTTTCCGCGCTCAATGTCTAACC
GTAGCGCAGTATCATGATCGGC
>ASV4
CGGTGACGATGCGCCCAGAGCCGTTTTCCGTAAGTCTGAAGACACCTGTTTCGCC
CATCTCGCAATTCCGAGCTGACACCCCTCCCAGCTTCCATTGTCGGCAGACCCCG
TCACCATGGGTATGCATTCTTCCCCGCGAGCCTCCCTGTCGCGCGCAGCCAATTC
TAACCCCTCCGCAGTATCATGATCGGT
>ASV5
CGGTGACGATGCGCCCAGAGCCGTTTTTCGTAAGTCCAAAGACACCTGTTTCGCC
CATCTCGCAATCCCAAGCTGACACTCTTCCTAGCTTCCATTGTCGGCAGACCCCG
TCACCATGGGTATGCATTCTCTCCGCGAGCCTCCCCATCGCGCACAGCCAGTTCT
AACCCCTCCGCAGTATCATGATCGGT
>ASV6
CGGTGACGATGCGCCCAGAGCCGTTTTCCGTAAGTCCAAAGACACCTGTTTCGCC
CGTCTCGCAATCCCGAGCTGACACTCTTCCCAGCTTCCATTGTCGGCAGACCCCG
TCACCATGGGTATGCATTCTCTCCGCGAGCCTCCCCATCGCGCACAGCCAGTTCT
AACCCCTCCGCAGTATCATGATCGGC
>ASV8
CGGTGACGATGCGCCCAGAGCCGTTTTCCGTAAGTCTAAAGACACCTGTTTCGCC
CGCACCAGAATCCCGAGCTGACACCCTCTGTAGCTTCCATTGTCGGCAGACCCCG
TCACCATGGGTATGCAATCTCCCTACGAACCTCCAATGCGCGCTCATTCAAATCT
AACCCCGGCGCAGTATCATGATCGGT
>ASV9
CGGTGACGATGCGCCCAGAGCCGTTTTCCGTAAGTCCAAAGACACCTGTTTCGCC
CGTCTCGCAATCCCGAGCTGACACTCTTCCCAGCTTCCATTGTCGGCAGACCCCG
TCACCATGGGTATGCATTCTCTCCGCGAGCCTCCCCGTCGCGCACAGCCAGTTCT
AACCCCTCCGCAGTATCATGATCGGC
>ASV10
CGGTGACGATGCGCCCAGAGCCGTTTTCCGTAAGTCTAAAGACACCTGTTTCGCC
CATCTCGCAATTCCGAGCTGACACTCTTCCCAGCTTCCATTGTCGGCAGACCCCG
TCACCATGGGTATGCATTCCCCCCGCGAGCCTCTTTATCGCGCGCAGCCAGTTCT
AACCCCTCCGCAGTATCATGATCGGT
>ASV11
CGGTGACGATGCGCCCAGAGCCGTTTTCCGTAAGTCTAAAGACACCTGTTTCGCC
CATCTCGCAATTCCGAGCTGACACTCTTCCCAGCTTCCATTGTCGGCAGACCCCG

TCACCATGGGTATGCATTCTCCCCGCGAGCCTCCCTGTCGCGCGCAGCCAATTCT
AACCCCTCCGCAGTATCATGATCGGT
>ASV15
CGGTGACGATGCGCCCAGAGCCGTTTTCCGTAAGTCTGAAGACACCTGTTTCGCC
CGCTCCAGAATCCCCAGCTGACGGCAATCTTAGCTTCCATTGTCGGCAGACCCCG
TCACCATGGGTATGCATCCTCCCCGCGAGCCTCCCCGTCGCGCGCAACCAGTTCT
AACCCCTCCACAGTATCATGATCGGT
>ASV16
CGGTGACGATGCGCCCAGAGCCGTTTTCCGTAAGTCTAAAGACACCTGTTTCGCC
CATCTCGCAATTCCGAGCTGACACTCTTCCCAGCTTCCATTGTCGGCAGACCCCG
TCACCATGGGTATGCATTCTCCCCGCGAGCCTCCCTGTCGCGCGCAGCCAGTTCT
AACCCCTCCGCAGTATCATGATCGGT
>ASV17
CGGTGACGATGCGCCCAGAGCCGTTTTCCGTAAGTCCAAAGACACCTGTTTTGCC
CATCTCGCAATCCCAAGCTGACACTCTTCCTAGCTTCCATTGTCGGCAGACCCCG
TCACCATGGGTATGCATTCTCTCCGCGAGCCTCCCCATCGCGCACAGCCAGTTCT
AACCCCTCCGCAGTATCATGATCGGT
>ASV18
CGGTGACGATGCGCCCAGAGCCGTTTTCCGTAAGTCTAAAGACACCTGCTTCGCC
CGCGCCAGAATCCCGAGCTGACACCCTCTCTAGCTTCCATTGTCGGCAGACCCCG
TCACCATGGGTATGCATTCTCCCCGCGAGCCTCCCATTCGCGCTCATTCAAATCT
AACCCCGGCACAGTATCATGATCGGT
>ASV20
CGGTGACGATGCGCCCAGAGCCGTTTTCCGTAAGTTTGAAGACACCTGTTTCGCC
CCGCCTAGAACTTCCAGCTGACAGTAACATAGCTTCCATTGTCGGCAGACCCCGT
CACCATGGGTATGCCCTCTTCCCGCAATTTCCGCCGTCGAGTTCAGAGATCTAAC
AATGGCGCAGTATCATGATCGGT
>ASV21
CGGTGACGATGCGCCCAGAGCCGTTTTCCGTAAGTCTGAAGACACCTGTTTCGCC
CGTCTCGCAATTCCGAGCTGACACCCCTCCCAGCTTCCATTGTCGGCAGACCCCG
TCACCATGGGTATGCATTCTCCCCGCGAGCCTCCCTGTCGCGCGCAGCCAATTCT
AACCCCTCGGCAGTATCATGATCGGT
>ASV23
CGGTGACGATGCGCCCAGAGCCGTTTTCCGTAAGTCTGAAGACACCTGTTTCGCC
CGTGCCAGAATTCCGAGCTGACACCACCCTAGCTTCCATTGTCGGCAGACCCCGT
CACCATGGGTATGCATCCTCCCCACCTCGCCCGTTTCCGCGCTCAATGTCTAACC
GTAGCGCAGTATCATGATCGGC
>ASV29
CGGTGACGATACGCCCAGAGCCGTTTTCCGTAAGTCCAAAGACACCTGTTTCGCC
CATCTCGCAATCCCAAGCTGACACTCTTCCTAGCTTCCATTGTCGGCAGACCCCG
TCACCATGGGTATGCATTCTCTCCGCGAGCCTCCCCATCGCGCACAGCCAGTTCT
AACCCCTCCGCAGTATCATGATCGGT
>ASV30
CGGTGACGATGCGCCCAGAGCCGTTTTCCGTAAGTCTGAAGACACCTGTTTCGCC
CGTGCCAGAATTCCGAGCTGACACCATCCTAGCTTCCATTGTCGGCAGTCCCCGT
CACCATGGGTATGCATCCTCCCCACCTCGCCCGTTTCCGCGCTCAATGTCTAACC
GTAGCGCAGTATCATGATCGGC
>ASV31
CGGTGACGATGCGCCCAGAGCCGTTTTCCGTAAGTCTAAAGACACCTGTTTCGCC
CGCGCCAGAATCCCGAGCTGACACCCTCTCTAGCTTCCATTGTCGGCAGACCCCG

TCACCATGGGTATGCATTCTCCCCGCGAGCCTCCCATTCGCGCTCATTCAAATCT
AACCCCGGCGCAGTATCATGATCGGT
>ASV33
CGGTGACGATGCGCCCAGAGCCGTTTTCCGTAAGTCCAAAGACACCTGTTTCGCC
CGTCTCGCAATCCCGAGCTGACACTCTTCCCAGCTTCCATTGTTGGCAGACCCCG
TCACCATGGGTATGCATTCTCTCCGCGAGCCTCCCCATCGCGCACAGCCAGTTCT
AACCCCTCCGCAGTATCATGATCGGC
>ASV35
CGGTGACGATGCGCCCAGAGCCGTTTTCCGTGAGTCCAAAGACACCTGTTTCGCC
CGTCTCGCAATCCCGAGCTGACACTCTTCCCAGCTTCCATTGTCGGCAGACCCCG
TCACCATGGGTATGCATTCTCTCCGCGAGCCTCCCCATCGCGCACAGCCAGTTCT
AACCCCTCCGCAGTATCATGATCGGC
>ASV37
CGGTGACGATGCGCCCAGAGCCGTTTTCCGTAAGTCTGAAGACACCTGTTTCGCC
CGCGCCAGAATCCCGAGCTGACACCTTCTCTAGCTTCCATTGTCGGCAGACCCCG
TCACCATGGGTATGCATTCTCCCCGCGAGCCTCCCCTTCGCGCTCATTAACTAAC
CCCGGCGCAGTATCATGATCGGT
>ASV38
CGGTGACGATGCGCCCAGAGCCGTTTTCCGTAAGTCTAAAGACACCTGTTTCGCC
CATCCCGCAATTCCGAGCTGACACCCATCTTAGCTTCCATTGTCGGCAGACCCCG
TCACCATGGGTATGCATTTCCCCCTGCGAACCTCCCTATCGCGCTCAACCATGTCT
AACCCCGGCGCAGTATCATGATCGGT
>ASV39
CGGTGACGATGCGCCCAGAGCCGTTTTCCGTAAGTCTGAAGACACCTGTTTCGCC
CGCTCCAGAATCCCCAGCTGACGGCAATCTTAGCTTCCATTGTCGGCAGACCCCG
TCACCATGGGTATGCATCCTCCCCGCGAGCCTCCCCATCGCGCGCAACCAGTTCT
AACCCCTCCACAGTATCATGATCGGT
>ASV41
CGGTGACGATGCGCCCAGAGCCGTTTTCCGTAAGTCTGAAGACACCTGTTTCGCC
CATGCCACATTTCCGAGCTGACACCCATCCTAGCTTCCATTGTCGGCAGACCCCG
TCACCATGGGTATGCATTCCCCCTCTCCCGCGCTCATTCCCGCGCGCACGCAATA
TCTAACTCTGGCGCAGTATCATGATCGGT
>ASV42
CGGTGACGATGCGCCCAGAGCCGTTTTCCGTAAGTCTAAAGACACCTGTTTCGCC
CGCGCCAGAATCCCGAGCTGACACCCTCTCTAGCTTCCATTGTCGGCAGACCCCG
TCACCATGGGTATGCATTCTCCCCACGAGCCTCCCATTCGCGCTCATTCATGTCTA
ACTCCGGCGCAGTATCATGATCGGT
>ASV44
CGGTGACGATGCGCCCAGAGCCGTTTTCCGTAAGTCTGAAGACACCTGTTTCGCC
CGTGCCAGAATTCCGAGCTGACACCATCCCAGCTTCCATTGTCGGCAGACCCCGT
CACCATGGGTATGCTTCCTCCTCACCTCGCCCGTTTCCGCGCTCAATGTCTAACCG
CAGCGCAGTATCATGATCGGT
>ASV45
CGGTGACGATGCGCCCAGAGCCGTTTTCCGTAAGTCTAAAGACACCTGTTTCGCC
CGCGCCAGAACCCCGAGCTGACACCCTCTCTAGCTTCCATTGTCGGCAGACCCCG
TCACCATGGGTATGCATTCTCCCCGCGAGCCTCCTTGTCGCGCTCATTCAAGTCTA
ACCCCGGCGCAGTATCATGATTGGT
>ASV47
CGGTGACGATGCGCCCAGAGCCGTTTTCCGTAAGTTCCCACCACCTGCGCTCTTC
ACCCACCGGACCACTCGGCTGACCACCTCTCAGCTTCCATTGTCGGCAGACCCCG

TCACCATGGGTATGCATCCTCCCCGCGCCCTCCGCCGAGGCCACCCCGTTCTAAC
CGCAGCGCAGTATCATGATCGGT
>ASV50
CGGTGACGATGCGCCCAGAGCCGTTTTCCGTAAGTCTGAAGACACCTGTTTCGCC
CGCCCATGAATCCCGAGCTGACACCCATCCCAGCTTCCATTGTCGGCAGACCCCG
TCACCATGGGTATGCATTCTCCCTGCGAACCCCTTCACCGCGCGCAGCCAATTCT
AATTCCTCCGCAGTATCATGATCGGT
>ASV52
CGGTGACGATGCGCCCAGAGCCGTTTTCCGTAAGTCTGAAGACACCTGTCTCACC
CCTCCAGAACTCTGAGCTGACAGCATTCTAGCTTCCATTGTCGGCAGACCCCGTC
ACCATGGGTATGCATTCTCCCAATCCCATTTCGCGCTCAATGTCTAACCGCAGCG
CAGTATCATGATCGGT
>ASV54
CGGTGACGATGCGCCCAGAGCCGTTTTCCGTAAGTCTGAAGACACCTGTTTCGCT
CGTGCCAGAATTCCGAGCTGACACCATCCTAGCTTCCATTGTCGGCAGACCCCGT
CACCATGGGTATGCATCCTCCCCACCTCGCCCGTTTCCGCGCTCAATGTCTAACC
GCAGCGCAGTATCATGATCGGT
>ASV58
CGGTGACGATGCGCCCAGAGCCGTTTTCCGTAAGTCCAAAGACACCTGTTTCGCC
CATCTCGCAATCCCAAGCTGACACTCTTCCTAGCTTCCATTGTCGGCAGATCCCG
TCACCATGGGTATGCATTCTCTCCGCGAGCCTCCCCATCGCGCACAGCCAGTTCT
AACCCCTCCGCAGTATCATGATCGGT
>ASV63
CGGTGACGATGCGCCCAGAGCCGTTTTCCGTAAGTCTACAGACACCTGTCCCACC
AACCGCAGAATTCCGAGCTGACAACCTCTTAGCTTCCATTGTCGGCAGACCCCGT
CACCATGGGTATGCACTCTCCCCACGCTCATTGTCGCGCTCAATGTCTAACCGCA
GCGCAGTATCATGATCGGT
>ASV64
CGGTGACGATGCGCCCAGAGCCGTTTTCCGTAAGTCTGAAGACACCTGTCTCACC
CGTCTCGAGAATTCCGAGCTGACACCACCCTAGCTTCCATTGTCGGCAGACCCCG
TCACCATGGGTATGCAGTTCCCCAAGCCCCATTGTTCGCGCTCAATCCTCTAACC
CCAGCGCAGTATCATGATCGGT

Data S3 DNA sequence reads of the TEF gene assigned to *Cladosporium*

>ASV1
TGAGCCTCACTCCGGCACGATGCTATCTCTTCGCGACGTTTCCTCCCTCTGCCCCG
CCATGACACCCCGCCTCGTCGCAATTTGCGATAAGGATGTGGGCCGCTCTGGCTT
GGCATGGACATACACAACGACGAGATAGCATTGCCACACAACATCACTACGTAC
AAATTGCTGACAACCACAATAGGAAGCCGCCGAACTC
>ASV2
TAAGCACTATTCCCGTGGTGCTCTCTTCCCCATCGCGGTGTTTTGCCCCTCTGTCC
CGCCACGCCACCCCACCTCGTCGCAATCTGCGATAAGGTGCGGTACACCGGCTCG
GCTTGGCATGGGCTGCACTACCATGGAGATACCATCGCCACGCATTCGTCACATC
ACAACACACTGCTAACAATCACTATAGGAAGCCGCCGAACTC
>ASV4
TGAGCACCGCTCCGGCACCATGTCATCTCTTCGCGATGTTTCATCCCTCTGCCCCG
CCACGTCACCCCACCTCGTCGCAAATTTGCGATAAGGATGTGGGACACCCTGGCT
TGGCACGGATGTGCACCACGACAGATGGCATCGCCACACAACAGCGCTCGTCGA
GCACATCACTGACAACCATAATAGGAAGCCGCCGAACTC
>ASV5
TGAGCATCACTCGGGCACATTGTCACCTTGTCGCGATGGCATATCCCTCTGCCCC
GCCACTACACCCCGCCTCGTTGCAAATTTGCGATAAGGATGTGGGCCGCCCTGGC
TTGGCACGGACGTCCATCACGACAAGACAGCATTGCCACACAAACCATACCAAC
AGAACGGATAACTGACAACCACCATAGGAAGCCGCCGAACTC
>ASV6
TGAGCATCACTCGGGCACATTGCCACCTTGTCGCGATGACTTATTCCTCTGCCCC
GCCAAATCACCCCGCCTCGTCGCAAATTTGCGATAAGGATGTGGGACGCCCTGG
CTTGGCATGGACAACTGTCGCGACAAGATAGCATCGCCACTCAACACATCTCTCC
GGCACATCACTGACAACCGCAATAGGAAGCCGCCGAACTC
>ASV8
TGAGCATCACTTCGGCACCATGTCATCTCTTCGCGATGTTTCATCCCTCTGCCCCG
CCACGTCACCCCACCTCGTCGCAAATTTGCGATAAGGATGTGGGACACCCTGGCT
TGGCATGGATGTGCACCACGAGAGAAGGCATCGCCACACAACAGCGCTCGTCGA
GCACATCACTGACAACCACAATAGGAAGCCGCCGAACTC
>ASV9
TGAGCATCACTCGGGCACAATGTCACCTTGTTGCGATGGCACATCCCTCTGCCCC
GCCACAACACCCCGCCTCGTCGCAAATTTGCGATAAGGATGTGGGCCGTCCTGGC
TTGGCATGGAGGCGCCTCGCGACAAGACAGCATTGCCACTCATTACGCCTCACA
GAGCACATCACTGACAACCACAATAGGAAGCCGCCGAACTC
>ASV10
TGAGCCTTACTTCACGGCAATGGCGTCCTCTTCGCAGTGTTGTACCCCTCCGCCCC
ACCGCGCGCCACCCCACCTCGTCGCAATCTGCGATAAGATGTGGGACACGACTT
GGCCGGGCACGGACAACACTGCGATGAAGACTTCATCGCCACACGCCATCACCA
TCACAATACATTGCTAAAAACCCTCCCAGGAAGCCGCTGAACTC
>ASV11
TGAGCATCACTCGGGCACATTGCCACCTTGTCGCGATGACTTATCCCTCTGCCCC
GCCTCAACACCCCGCCTCGTCGCAAATTTGCGATAAGGATGTGGGACGCCCTGGC
TTGGCACGGACAACCGTTGCGACAAGACAGCAACGCCACTCAACCCACCTCTCC
GAGCACATCACTGACAGCCGCAATAGGAAGCCGCCGAACTC
>ASV13
TGAGCATCACTCGGGCACAATGCTGTCTTGTTACGATGGCACATCCCTCTGCCTC
GCCACAACACCCCGCCTCGTCGCAAATTTGCGATAAGGATGTGGGCCGTCCTGGC

TTGGCATGGAGGCGTCTCGCGACAAGACAGCATTGCCACTCAACACGCCTCACA
GAGCACATCACTGACAACCGCAACAGGAAGCCGCCGAACTC
>ASV14
TAAGCACTACTCCCGTGGTGCTCTCTTCCCATCGCGGTTTTTGCCCCTCTGTCC
CGCCACGCCACCCCACCTCGTCGCAATCTGCGATAAGGTGCGGTACACCGGCTTG
GCTTGGCATGGGCTGCACTGCCATGGAGATACCATCGCCACTCATCCGCCACACA
ACAACTCATTGCTAACAACCACCATAGGAAGCCGCCGAACTC
>ASV15
TGAGCATCACTCGGCCACATTGCCACCTTGTTGCGATGACTTATCCCTCTGCCCC
GCCACAACACCCCGCCTCGTCGCAAATTTGCGATAAGGATGTGGGACGCCCTGG
CTTGGCATGGACAACCGTCGCGACAAGACAGCATCGCCACTCAACACATCTCTCC
GGCACATCACTGACAACCGCAATAGGAAGCCGCCGAACTC
>ASV16
TGAGCATCACTTGGGCACAATGCCACCTTGTCGCGATGAGTTATCCCTATGCCCC
GCCACAACACCCCGCCTCGTCGCAAATTTGCGATAAGGATGTGGGCCGCCCTGG
CTTGGCATGGACCAGCACCGCGACAAGATGGCATCGCCACAACACTGTTCACAA
TGACCACATCACTGACATCTACAACAGGAAGCCGCCGAACTC
>ASV17
TGAGCATCACTCGGGCACAATGCCACCTTGTCGCGATGGCACATCCCTCTGCCTC
GCCACAACACCCCGCCTCGTCGCAAATTTGCGATAAGGATGTGGGCCGCCCTGG
CTTGGCATGGACGTCCATCACGACAAGACAGCATTGCCACTCAACACGCCTATTA
TAGCACATCACTGACGACCGCAACAGGAAGCCGCCGAACTC
>ASV19
TGAGCATCACTCGGGCACAATGCCACCTTGTCGCGATGGCACATCCCTATGCCCC
GCCACAACACCCCGCCTCGTCGCAAATTTGCGATAAGGATGTGGGCCGCCCTGG
CTTGGCATGGACGTCCATCACGACAAGACAGCATTGCCACTCAACACGCCTTTCA
TAGCACATCACTGACGACCGCAACAGGAAGCCGCCGAACTC
>ASV20
TGAGCATCACTCGTGCACATTGTCACCTTGTCGCGATGGCACATCCCTCTGCCCC
GCCACCACACCCCGCCTCGTCGCAAATTTGCGATAAGGATGTGGGCCGGCCTGG
CTTGGCACGGACGTCCATCATGACGAGGCAGCATTGCCACACAAACCATATCAA
CAGAACGGATAACTGACAACCGCCATAGGAAGCCGCCGAACTC
>ASV23
TGAGCATCACTTTGGCACACTGTCGCCTTGTTGCGATGGCACATCCCTCTGCCCC
GCCACAGCACCCCGCCCTCGTCGCAAATTTGCGATAAGGATGTGGGCCGCCCTG
GCTTGGCACGGACGTCCATCACGACGAGGCAGCATTGCCACACAAACCATACTA
TCAGAAACAGATAATTGACAACCACCATAGGAAGCCGCCGAACTC
>ASV24
TAAGCCTCACTCCAGTACAGCGATATCTCATCGCGATGTTTCCTCCCCCTGCCCC
GCCATGACACCCCGCCTCGTCGCAATTTGCGATAAGGATGTGGGCCGCCCTGGCT
TGGCATGGACCACCATCGCAACAGGACATCATTGTCATTCAACATCACTCCACAA
ACACATGGCTGACAACCACAACAGGAAGCCGCCGAACTC

Supplementary References

1. White TJ, Bruns T, Lee S, Taylor J. Amplification and direct sequencing of fungal ribosomal RNA genes for phylogenetics. In: Innis MA, Gelfand DH, Sninsky JJ, White TJ, editors. PCR protocols: a guide to methods and applications. London: Academic; 1990. pp. 315-22.
2. Carbone I, Kohn LM. A method for designing primer sets for speciation studies in filamentous ascomycetes. Mycologia. 1999;91(3):553-6. https://doi.org/10.2307/3761358.
